# Human umbilical cord-derived mesenchymal stem cells and auto-crosslinked hyaluronic acid gel complex for treatment of intrauterine adhesion

**DOI:** 10.18632/aging.205704

**Published:** 2024-04-01

**Authors:** Jiaying Fan, Jingying Xie, Yunsheng Liao, Baoyu Lai, Guixin Zhou, Wenqin Lian, Jian Xiong

**Affiliations:** 1 Department of Obstetrics and Gynaecology, Guangzhou Women and Children’s Medical Center, Guangzhou Medical University, Guangzhou 510623, Guangdong, China; 2 Department of Surgery, Guangzhou Women and Children’s Medical Center, Guangzhou Medical University, Guangzhou 510623, Guangdong, China

**Keywords:** human umbilical cord-derived mesenchymal stem cells, autocrosslinked hyaluronic acid gel, intrauterine adhesion, preclinical study, endometrial regeneration

## Abstract

Objective: The purpose of this study was to explore the therapeutic characteristics of mesenchymal stem cells generated from human umbilical cord (hUC-MSCs) when utilized in conjunction with auto-crosslinked hyaluronic acid gel (HA-gel) for the management of intrauterine adhesion (IUA). The goal was to see how this novel therapy could enhance healing and improve outcomes for IUA patients.

Methods: In this study, models of intrauterine adhesion (IUA) were established in Sprague-Dawley (SD) rats, which were then organized and divided into hUC-MSCs groups. The groups involved: hUC-MSCs/HA-gel group, control group, and HA-gel group. Following treatment, the researchers examined the uterine cavities and performed detailed analyses of the endometrial tissues to determine the effectiveness of the interventions.

Results: The results indicated that in comparison with to the control group, both HA-gel, hUC-MSCs, and hUC-MSCs/HA-gel groups showed partial repair of IUA. However, in a more notable fashion transplantation of hUC-MSCs/HA-gel complex demonstrated significant dual repair effects. Significant outcomes were observed in the group treated with hUC-MSCs and HA-gel, they showed thicker endometrial layers, less fibrotic tissue, and a higher number of endometrial glands. This treatment strategy also resulted in a significant improvement in fertility restoration, indicating a profound therapeutic effect.

Conclusions: The findings of this study suggest that both HA-gel, hUC-MSCs, and hUC-MSCs/HA-gel complexes have the potential for partial repair of IUA and fertility restoration caused by endometrium mechanical injury. Nonetheless, the transplantation of the hUC-MSCs/HA-gel complex displayed exceptional dual healing effects, combining effective anti-adhesive properties with endometrial regeneration stimuli.

## INTRODUCTION

Asherman syndrome, also known as intrauterine adhesion (IUA), arises because of the basal layer of the endometrium which results in a partial or complete blockage of the uterine cavity. This condition causes hypomenorrhea, amenorrhea, infertility, miscarriage, and abnormal placentation [1]. Primary risk factors for developing IUA include surgical procedures such as repeated hysteroscopic surgeries, dilation, and curettage (D&C) as well as induced abortions and myomectomies damage [2, 3]. Women in the reproductive age group are particularly susceptible to reproductive disadvantage due to severe endometrial dysfunction. IUA are widely acknowledged as the second most prevalent factor contributing to female infertility, with fallopian tube obstruction being the sole reason that surpasses it in significance [4, 5].

The goal of IUA therapy is not only to restore the uterine cavity’s structure but, more critically, the patient’s endometrial function and ability to conceive. Current treatment strategies encompass hysteroscopic adhesion resection, postoperative adhesion prevention and promotion of endometrial regeneration [6]. The use of physical barriers, including contraceptive devices and intrauterine balloon devices including The Foley balloon and INTERCEED® (an absorbable barrier made of oxidized regenerated cellulose), as well as semi-solid barriers such as hyaluronic acid, have been shown to reduce the likelihood of adhesions reforming following surgery [7–11]. Additionally, enhancing vascular supply to the endometrium following hysteroscopic adhesiolysis, through methods such as amnion grafts and postoperative hormone therapy using estrogen with or without progestin, plays a crucial impact on endometrial repair and regeneration [6, 12–14]. While some cases have had excellent results, patients with moderate to severe adhesion generally have a poor prognosis. Currently, the main challenge in treating IUA is overcoming endometrial fibrosis, which causes a high rate of adhesion recurrence (up to 66% after the initial surgery) [6] and a low live birth rate [15].

Generally, promoting endometrial repair and regeneration while using stem cell-based therapy is getting increased recognition as an approach for specifically addressing tissue damage as well as fibrosis [16]. Among a high variety of sources of stem cells, mesenchymal stem cells produced from the human umbilical cord (hUC-MSC expressing intermediate differentiation potential between embryonic and adult stem cells [16] are the most used for tissue regeneration and cell therapy. The reason why they are being used in this increased fashion is because they are easily harvested and collected from discarded umbilical cords and have high proliferation, strong differentiation, and migration abilities as well as weak immunogenicity and non-tumorigenicity [17, 18]. Previous research has demonstrated that hUC-MSC can develop into epithelial and endometrial stromal cells [17] supporting their potential of restoring damaged endometrial structure and function. This includes development of the endometrial morphology, angiogenesis, and cell proliferation, which has established hUC-MSC transplantation’s efficiency in treating IUA [19].

Intrauterine injection is considered the most effective method for hUC-MSC transplantation, nonetheless, optimizing this procedure to maintain the cell viability and to promote endometrial regeneration remains quite a challenge. Accordingly, intrauterine transplantation of hUC-MSC, combining a variety of scaffolds (amniotic matrix, electrospun fiber films, collagen, hydrogels, and sponges), has been applied and therefore have proved to improve the therapeutic effect of IUA [20–24]. However, there is still no consensus on the best material to use. Selecting the appropriate material during intrauterine transplantation remains crucial to better prolong hUC-MSC retention and perform its activity of endometrial regeneration.

China Food and Drug Administration (CFDA) has approved auto-cross-linked hyaluronic acid gel (HA-gel), as an innovative medical device for adjunctive prevention of adhesion recurrence after hysteroscopic adhesiolysis, another potential semi-solid barrier which is designed to mimic the natural properties of synovial fluid and extracellular matrix. HA-gel has the advantages of few degradations and high viscosity by using an auto crosslinking technique to activate and modify linear hyaluronan molecules into a three-dimensional web-like structure. This technique was developed to extend the gel’s half-life, which leads to a prolonged absorption time (up to 7 to 14 days). Additionally, its other material features can enhance the quality of the endometrium and uterine receptivity. In addition, the gel’s ability to expand ensures continuous isolation of the uterine cavity following surgery, effectively reducing the risk of re-adhesion [25].

The efficacy of HA-gel in preventing IUAs was reported by the American Association of Gynecologic Laparoscopists and the European Society of Gynecological Endoscopy in 2017 [6]. This discovery has been corroborated by many experimental and clinical investigations [11, 26]. Furthermore, a naturally occurring mixture of extracellular matrix and synovial fluid has been identified as another potentially effective semi-solid barrier for this application. However, randomized clinical trials (RCT) show opposite results, implying that HA-gel did not improve IUA recurrence following hysteroscopic adhesiolysis [27].

In this study, we evaluated the fine compatibility with hUC-MSC and HA-gel through a series of *in vitro* tests, including verification of hUC-MSCs, safety assessments, and cytogenetic evaluations of HA-gel. Additionally, we developed a complex of hUC-MSCs loaded on HA-gel in a rat IUA model to increase local stem cell activity and perseverance in endometrial regeneration. Our findings show that this combined approach is more effective than either treatment alone in terms of increasing endometrial thickness, gland count, and successful pregnancy rates.

## MATERIALS AND METHODS

### hUC-MSCs preparation

Frozen hUC-MSCs from passages (P) 4 to P8 were thawed and cultivated in DMEM with 1% penicillin/streptomycin (Gibco, USA) and 10% (v/v) fetal bovine serum (Gibco, USA) under conditions suitable for cell growth (37° C and 5% CO_2_). After 48 hours, upon reaching 80-90% confluence, cells were harvested by stem cell digestive juice (Gibco, USA) treatment for subculturing. Experiments utilized cells from both the fourth and eighth passages.

### hUC-MSCs culture and recovery on HA-gel

hUC-MSCs (1×105 cell per well) and 300 ul of HA-gel [Con., 5 mg/ml; Bioregen, Co., Ltd., China, approved by CFDA as a medical device (no. 20153641542)] were gently mixed evenly with sterile syringes in 24-well plates and then added DMEM (Gibco, USA), including 10% (v/v) FBS (FBS, Gibco, USA),1% penicillin/streptomycin (Gibco, USA). HA-gel was digested using hyaluronidase following a 48-hour co-culture and released hUC-MSCs, which were recovered and cultured for 48 hours at 37° C and 5% CO_2_ incubator. Afterwards, the cells were harvested using pancreatin digestion for use in future experiments.

### Phenotype characterization and identification of hUC-MSCs on an HA-gel

The morphological parameters of hUC-MSCs, mixed both with or without HA-gel, were observed at 0, 24, and 48 hours of culture. Meanwhile, Cell morphology was observed after culturing for 48 hours when hUC-MSCs (1×105 cells per well), released from HA-gel, were plated in 6-well plates. The representative pictures were captured using the Olympus CKX4 microscope (Olympus, Japan) after 24 and 48 hours post-culture. To assess the phenotype of the recovered hUC-MSCs, cell surface markers (CD90, CD105, CD34, CD45) were detected with fluorescence-activated cell sorting (FACS). To identify the differentiation potential of hUC-MSCs on HA-gel, the mesenchymal stem cells’ capabilities to promote osteogenesis, adipogenesis, and chondrogenesis were evaluated. Briefly, recovered hUC-MSCs (4×104 cells per well) were plated in 6-well plates and treated with specific induction media. Specifically, osteogenic induction medium (Invitrogen, USA) and adipogenic differentiation medium (Invitrogen, USA). Alizarin Red S (Invitrogen, USA) staining was used to confirm the positive induction of osteogenic differentiation after 21 days, and oil red O (Invitrogen, USA) staining was used to confirm the positive induction of adipogenic differentiation after 14 days. Chondrogenic induction was performed using the pellet method, and after 14 days of induction, Alcian Blue (Invitrogen, USA) and Safranin O (Invitrogen, USA) staining were applied for identification. This comprehensive strategy enabled a thorough evaluation of the hUC-MSCs’ multipotent differentiation capabilities.

### hUC-MSCs safety assessment on an HA-gel

To investigate the safety of hUC-MSCs on the HA-gel and to evaluate the viability of hUC-MSCs, trypan blue staining was performed. This approach enabled accurate counting of the live cell following isolation hUC-MSCs from HA-gel. The experiment was carried out in three replications to ensure reliability and consistency in results.

Cell proliferation was tested on hUC-MSCs and released hUC-MSCs from HA-gel. plates with 96 wells with DMEM and 1× 4 cells were sown per well, and cells were cultivated for 4 hours. The manufacturer’s directions assessed stem cell proliferation using the CCK-8 assay (Abcam, UK). The microplate reader measured optical density at 450 nm after 24, 48, and 72 hours. Eight complex holes were present in each group, and statistics were gathered for the average optical densities.

An Annexin V Apoptosis Detection Kit from Invitrogen in the United States was used to identify cell apoptosis. At 48 hours after co-culture, hUC-MSCs were collected and followed by hyaluronidase digestion. For control, hUC-MSCs were cultivated in DMEM with 10% FBS as the negative control and in water as a positive control. Cells were put in into Eppendorf tubes to undergo the process of centrifugation, for 5 minutes at 4° C while being given two PBS washes collecting 1~5× cells. In the following step, 5 μl of FITC-labeled annexin V in 100 μl of binding buffer, 15 minutes at room temperature, and 10 μl of PI were added.400 μl of binding buffer was added stirred and put on the ice following the anti-dark reaction. Data analysis was done using FACS.

Scratch assay was used to gauge the capacity of the cells to migrate. DMEM without FBS was used to seed cells on six-well plates, and they were allowed to develop until 90% of them were confluent. After cell adherence, the plates were incubated with serum-free medium in an incubator with 5% CO_2_ at 37° C as a step after being scratched with a sterile pipette tip to make straight wounds. The cells were examined using an Olympus (Japan) microscope at 0 and 16 hours.

### hUC-MSCs cytogenetic evaluation on an HA-gel

Telomerase activity of hUC-MSCs on an HA-gel was examined using a modified gel-telomerase repeated amplification technique (TRAP) test. The positive control was hUC-MSCs cultured with a regular medium, while as the negative control Hela cells and human skin fibroblasts were used, and the baseline was set to a TSR8 control. Using the TRAPeze telomerase detection kit in accordance with the manufacturer’s instructions, cells were screened for telomerase activity. The following step included the utilization of cells to be tested for telomerase activity using TRAPese telomerase detection kit, in accordance with the manufacturer’s instructions.

To evaluate the chromosome stability of the hUC-MSCs on an HA-gel, karyotype analysis was used to recover hUC-MSCs from HA-gel compared to hUC-MSCs undergoing similar analysis. Colchicine was briefly added to the culture 2 hours prior to the end of the process at a final concentration of 20 μg/ml. The cells were then collected and centrifuged for 10 minutes at 2000 rpm after being digested for 8 minutes with 2 cc of pancreatic enzymes and EDTA solution. In 8 ml of a hypotonic solution containing 0.075 M KCl, cell pellets were then resuspended, incubated for 30 min at 37° C in a water incubator, and then centrifuged for 10 minutes at 2,000 rpm after being fixed in 2 ml of a 3:1 methanol to the acetic solution. After further centrifugation, the supernatant was taken out, and 8 ml of ice-cold fixative solution was added. After being put onto a cold slide, Giemsa staining was applied after the cells had been trypsinized and treated at 75° C for three hours. Metaphase status image was captured using a Leica fully automated scanner (Leica, Germany), and karyotyping was analyzed with CytoVision software (Leica, Germany).

### Establishment of the IUA-induced rat model

Female Sprague-Dawley rats weighing 250±20g were purchased from the Guangdong Medical Experimental Animal Center with license number SCXK (Yue) 2018-0002. The rats were kept in a controlled environment in which we have maintained sterility, by ensuring the absence of contaminants. In addition, the rats were provided with unrestricted availability of food and water. The ethical utilization of animals in this study was approved by the Ethics Committee of Guangzhou Women and Children’s Medical Center.

To minimize individual differences and ensure reliable data, we used the random assignment method for grouping. The 40 female SD rats were numbered individually, and then the rats were randomly divided into 5 groups (8 rats each), including control (CON) group, IUA group, HA-gel group, hUC-MSCs group, and combined treatment (HA-gel+hUC-MSCs) group. Among 8 rats in each group, 1 was used for pathology testing, 3 for protein molecular testing, and 4 for *in vivo* imaging and fertility experiments.

The IUA model was established using mechanical injury and lipopolysaccharide (LPS) induction [28]. After administering chloral hydrate anesthesia, all rats were put on the operation table. A surgical procedure involved creating a 3 cm longitudinal cut along the central axis of the abdominal region to gain access to the uterus. Subsequently, a small incision of approximately 3 mm was made in the lower 1/3 portion of the uterus. A 2-3 mm diameter scraping spoon gently scraped and damaged the endometrium until the uterine wall became rough and semitransparent. Vascular forceps were employed to secure the ends of the uterus, while an LPS solution measuring 0.5 ml in volume was introduced into the uterine cavity. The solution was withdrawn after 5 minutes. Lastly, the uterus was sutured after LPS-soaked cotton thread was introduced into the uterine cavity. Following the cleansing of the abdominal cavity with a normal saline solution, before closure. After 24 hours of post-operation, the cotton thread was removed.

In the IUA group, no additional treatment was administered after modeling. Conversely, in the hUC-MSCs group, after modeling, 1 ml of normal saline solution containing 3×10^5 hUC-MSCs was injected into one side of the uterine horn. In the HA-gel group, an experimental treatment consisted of administering 1 mL of uterine protective gel. This was injected into one side of the uterine horn after the modeling procedure. In the HA-gel+hUC-MSCs group, 1 ml of uterine protective gel containing 3×10^5 hUC-MSCs was injected into one side of the uterine horn after modeling. During the treatment period, all groups except for the IUA group received oral administration of 0.026 mg estrogen daily as an adjuvant therapy. Rats were euthanized after 4 weeks of therapy, and endometrial tissues were excised and subsequently immersed in a 10% formaldehyde solution for fixation in preparation for subsequent analysis.

### Functional assessment of the uteri

7 days post-surgery and treatment injections, a subgroup of rats (n = 4) was transferred to separate cages, where they were paired with male rats for 96 hours to enable mating. After 10 days post-mating, the female rats were euthanized, and the number of fetuses on both uterine sides was counted.

### Hematoxylin-eosin staining

Rat endometrial tissues were dried in a graded series of alcohol, cleaned in xylene, and fixed in paraffin at 60° C to observe the histopathological changes. The pieces of the paraffin blocks were then flattened by floating them in a 42° C water bath after being sectioned (3 μm thick). The sections were then mounted on glass slides and incubated at 60° C overnight. The paraffin sections were successfully deparaffinized for 10 minutes in xylene I and II. Following deparaffinization, the sections were immersed for 2 minutes in 100%, 95%, and 75% ethanol, respectively. The specimens were subjected to a hematoxylin staining process for 3 minutes. This was followed by differentiation in a solution of 0.5% hydrochloric acid alcohol for 3 seconds, after which the specimens were washed with tap water for 10 seconds. The counterstaining procedure was performed using eosin for 5 minutes. Following this the sample was rinsed with tap water to remove any remaining dye and was dehydrated for two minutes in ethanol concentrations of 85%, 95%, and 100%. The sections were then immersed in xylene I and xylene II for 3 minutes each. To finalize the preparation, neutral resin was used to mount the sections, which were dried and stored. Three fields of view from each sample of uterine tissue were chosen for high-power microscopy examination.

The width of the uterine cavity was measured using ImageJ software, and the number of endometrial glands was recorded.

### Masson staining

To access the collagen deposition in rat endometrium, paraffin-embedded sections were first deparaffinized and hydrated. They were stained with Masson’s trichrome stain, rinsed quickly with a 2% acetic acid solution, then differentiated for 5 minutes with 5% phosphotungstic acid, soaked in a 0.2% acetic acid solution and stained for 5 minutes with a bright green staining solution, three times with 0.2% acetic acid solution, twice with 100% ethanol to dehydrate, and once with xylene to clear for 5 minutes, and finally sealed with neutral gum. Using the high-power microscope, 3 fields of view for each uterine tissue sample were examined. The ratio of collagen deposition within endometrial tissue was calculated using ImageJ software.

### Immunofluorescence assay

Tissues were fixed for 20 minutes in 95% alcohol. Following this, the sections were subsequently subjected to treatment with a solution of 0.5% Triton X-100 (Sigma, USA) at ambient temperature for a duration of 20 minutes. The samples were incubated for a total of one hour at a temperature of 37° C, in the presence of 10% normal goat serum obtained from Gibco, United States. Subsequently, the slides underwent treatment with primary antibodies targeting the human nuclear antigen (Abcam, USA) and vimentin (Abcam, USA) for an extended period at 4° C, following the initial blocking step. After that, the sections were treated with Alexa Fluor-conjugated secondary antibodies from the American company Thermo Fisher Scientific for an additional hour at room temperature. 4,6-diamidino-2-phenylindole (Life Technologies, USA) staining was performed to visualize the DNA. Finally, fluorescence microscopy images were captured using a Leica DMI6000B microscope completing the immunofluorescence procedure.

### Western blot

The bicinchoninic acid (BCA) assay was used to extract and quantify total protein lysates. Following this, a total of 50 μg of protein was applied onto a sodium dodecyl sulfate-polyacrylamide gel and subjected to electrophoresis. After the electrophoresis, the proteins were then transferred onto polyvinylidene fluoride membranes. To prevent non-specific binding, these membranes were incubated at room temperature for an hour in a solution containing 5% bovine serum albumin (BSA). Once the blocking step was completed, we conducted a two-hour incubation with primary antibodies against SDF-1 (1:300, Santa Cruz, USA), CXCR4 (1:500, Santa Cruz, USA), TAZ (1:300, Abcam, USA), and p-transcriptional co-activator with PDZ-binding motif, referred to as p-TAZ (1:800, CST, USA). The membranes were then incubated with a secondary antibody (1:1,000, Beyotime, China) that was conjugated to horse radish peroxidase. As a loading control, glyceraldehyde-3-phosphate dehydrogenase was utilized. Using the Tanon-5200 gel imaging system, the bands were visualized.

### Statistical analysis

Statistical analyses were conducted using SPSS software (version 22.0 IBM, USA). The mean ± standard deviation for continuous variables was presented. A one-way analysis of variance (ANOVA) was employed to compare group means, followed by the LSD test for between-group comparisons. Analyzing the outcomes of fertility tests was done using the 2. One, two, and three asterisks, respectively, indicate that the significance levels of P< 0.05, P < 0.01, and P < 0.001, respectively. A significance threshold of P < 0.05 was employed.

### Data availability

Upon reasonable request, the data will be made available to interested parties.

### Consent for publication

All authors have granted their permission for the publication of identifiable information, including the text, figures, and other materials, contained in this manuscript.

## RESULTS

### Phenotype characterization and verification of hUC-MSCs on HA-gel

**Figure 1:**
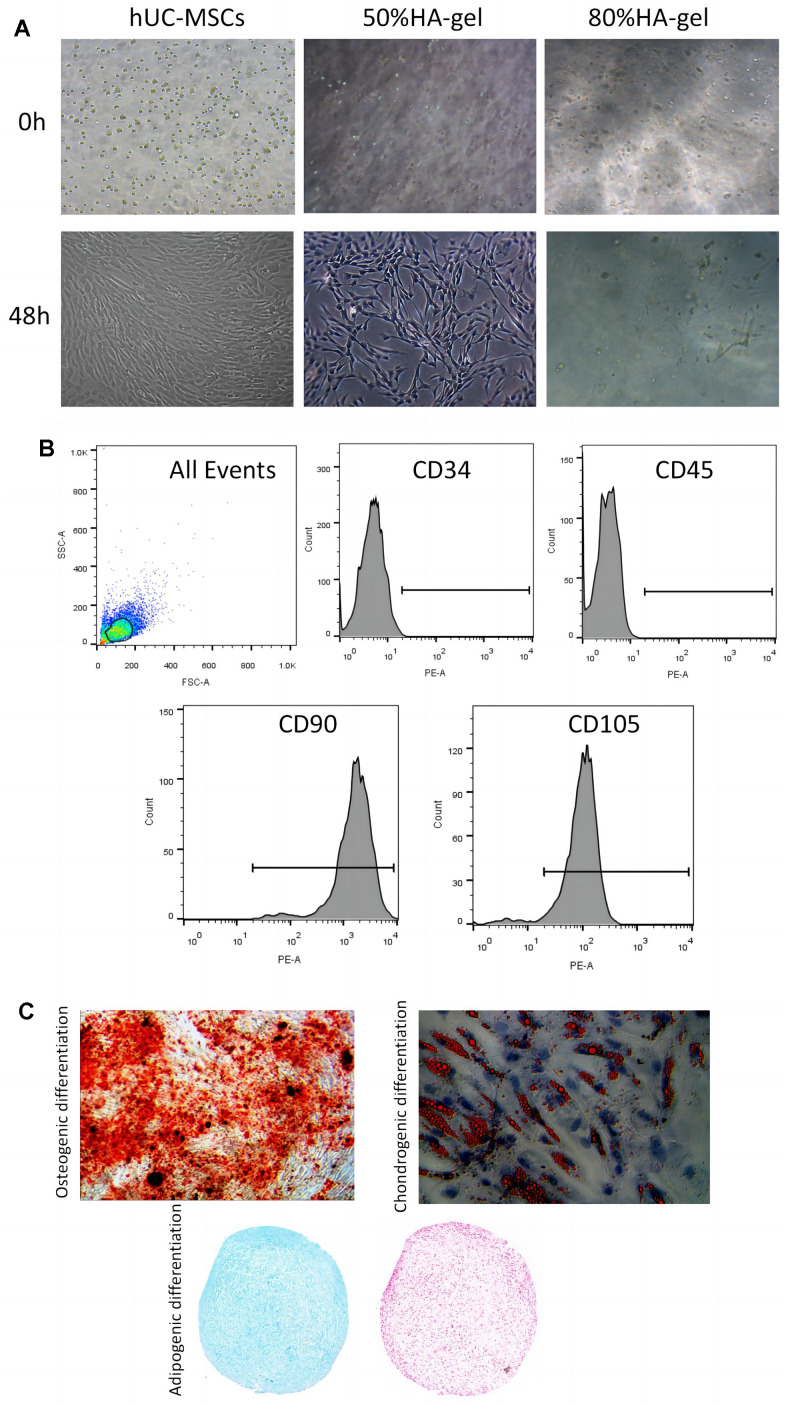
**hUC-MSCs characteristics and differentiation potential.** (**A**) The effect of HA-gel on the biological behavior of hUC-MSCs; (**B**) Surface markers characteristic of hUC-MSCs measured by flow cytometry; (**C**) The morphologic and intrinsic characterization of hUC-MSCs.

In the group treated with 50%HA-gel most of the cells were able to migrate out of the gel, and the migrated cells were able to adhere and grow well. In contrast, after 48 hours, in the 80%HA-gel group, the cells remained trapped within the gel forming a spherical form (Figure 1A). In addition, FACS results indicated that CD90 (99.80%) and CD105 (95.20%) were positive makers; CD34 (0.39%) and CD45 (0.10%) were the negative makers (Figure 1B). After being cultured in an enriching environment, the positively identified cells differentiated into osteoblasts, which initiated bone matrix formation, chondroblasts, which formed chondrogenic pellets within 21 days of induction, and adipocytes, which developed lipid droplets within 14 days of induction, as shown in Figure 1C.

### Safety assessment of hUC-MSCs on an HA-gel

**Figure 2:**
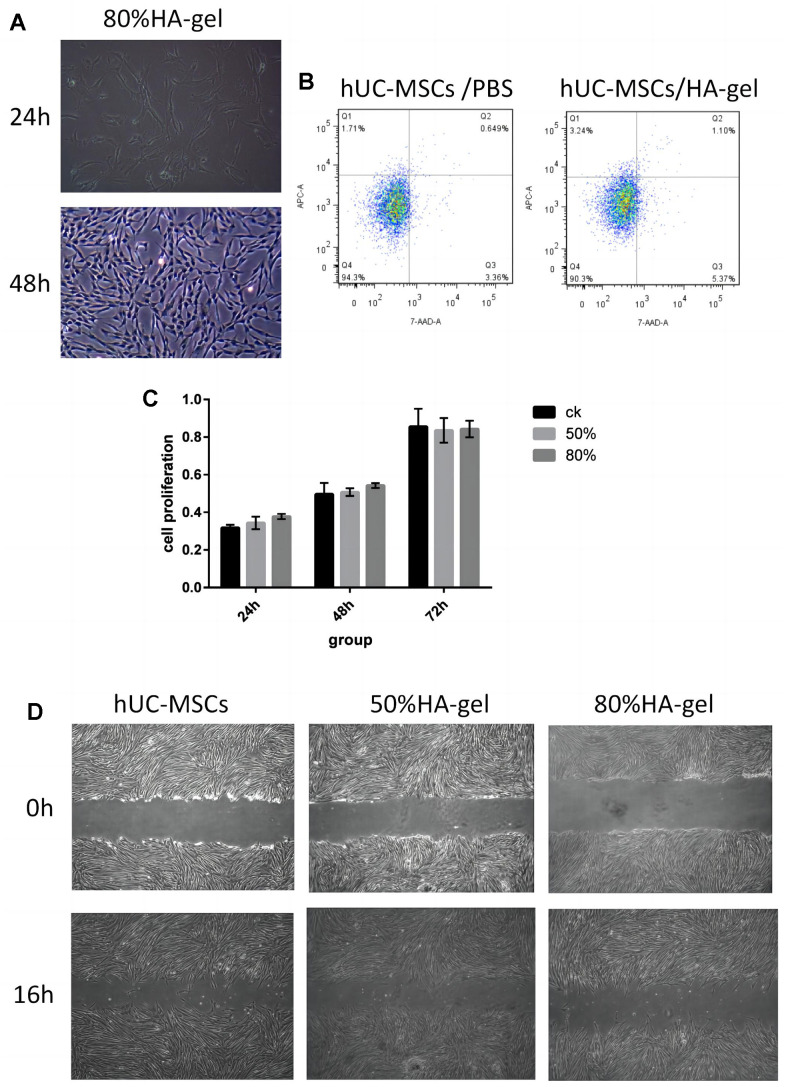
**Safety assessment and verification of hUC-MSCs on an autocrosslinked HA-gel.** (**A**) The influence of HA-gel on the morphology and adhesion of hUC-MSCs; (**B**) The influence of HA-gel on apoptosis of hUC-MSCs by FACS; (**C**) The effect of HA-gel on the proliferation ability of hUC-MSCs evaluated using CCK-8 assay; (**D**) The effect of HA-gel on the migration ability of hUC-MSCs evaluated by scratch assay.

The hUC-MSCs in the 50%HA-gel group were able to migrate out of the gel and exhibit average adherent growth. Hence there is no need to conduct further tests on their adhesive capabilities in this group. In the 80% HA-gel group, the condition of the cells was observed after culturing for 48h, and the gel was enzymatically digested to recover the cells for seeding into a 6-well plate. After that, the cells were cultured for 24 and 48 hours, and their morphology was observed to assess whether the gel had any impact on the adherent growth of MSCs. After 48 hours, the hUC-MSCs derived from the 80% HA-gel group still exhibited typical morphology characteristics of mesenchymal stem cells. They appeared spindle-shaped and showed adherent growth (Figure 2A). The flow cytometry study results revealed that compared to the PBS control group, the cells from the 50%HA-gel group did not exhibit a significant increase in necrotic, early, and late apoptotic cells (Figure 2B). The CCK-8 assay results indicated that after 48 hours of culture with 50% and 80% HA-gel, the hUC-MSCs did not significantly impact their growth and proliferation capability when returned to normal culture conditions (Figure 2C). The scratch assay results showed that both the 50% and 80% HA-gel did not significantly affect the migration ability of hUC-MSCs cells (Figure 2D).

### The effects of HA-gel on the genetic behavior of hUC-MSCs

**Figure 3:**
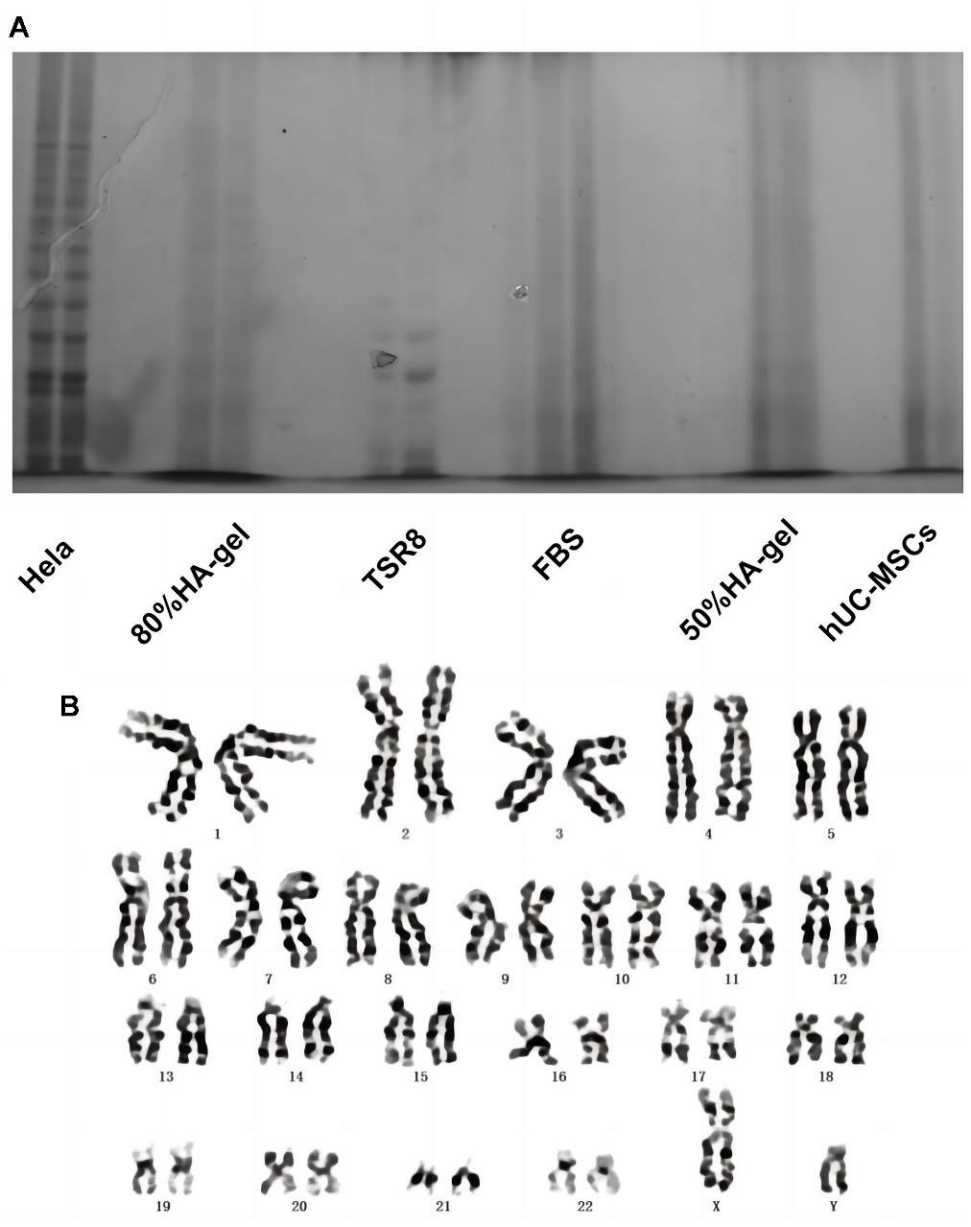
**The effects of HA-gel on the genetic behavior of hUC-MSCs.** (**A**) Telomerase activity detection; (**B**) Chromosomal karyotype analysis.

Figure 3A demonstrates that Hela cells exhibit significant telomerase activity. In comparison to Hela cells, the telomerase activity of hUC-MSCs cultured with 50% and 80% GA-GEL for 48 hours has shown markedly lower levels. This indicates that the telomerase activity of MSCs is not abnormally upregulated in the presence of HA-gel, which furthermore enhances that HA-gel-treated hUC-MSCs do not pose a risk of tumor formation. Additionally, the chromosomal structure’s specific morphology is exhibited with clarity in Figure 3B. After 48 hours of culture with 80% HA-gel, there is no apparent occurrence of deletions, duplications, inversions, or other abnormalities in the chromosomes of hUC-MSCs.

### The effect of HA-gel on hUC-MSCs in the IUA model

**Figure 4:**
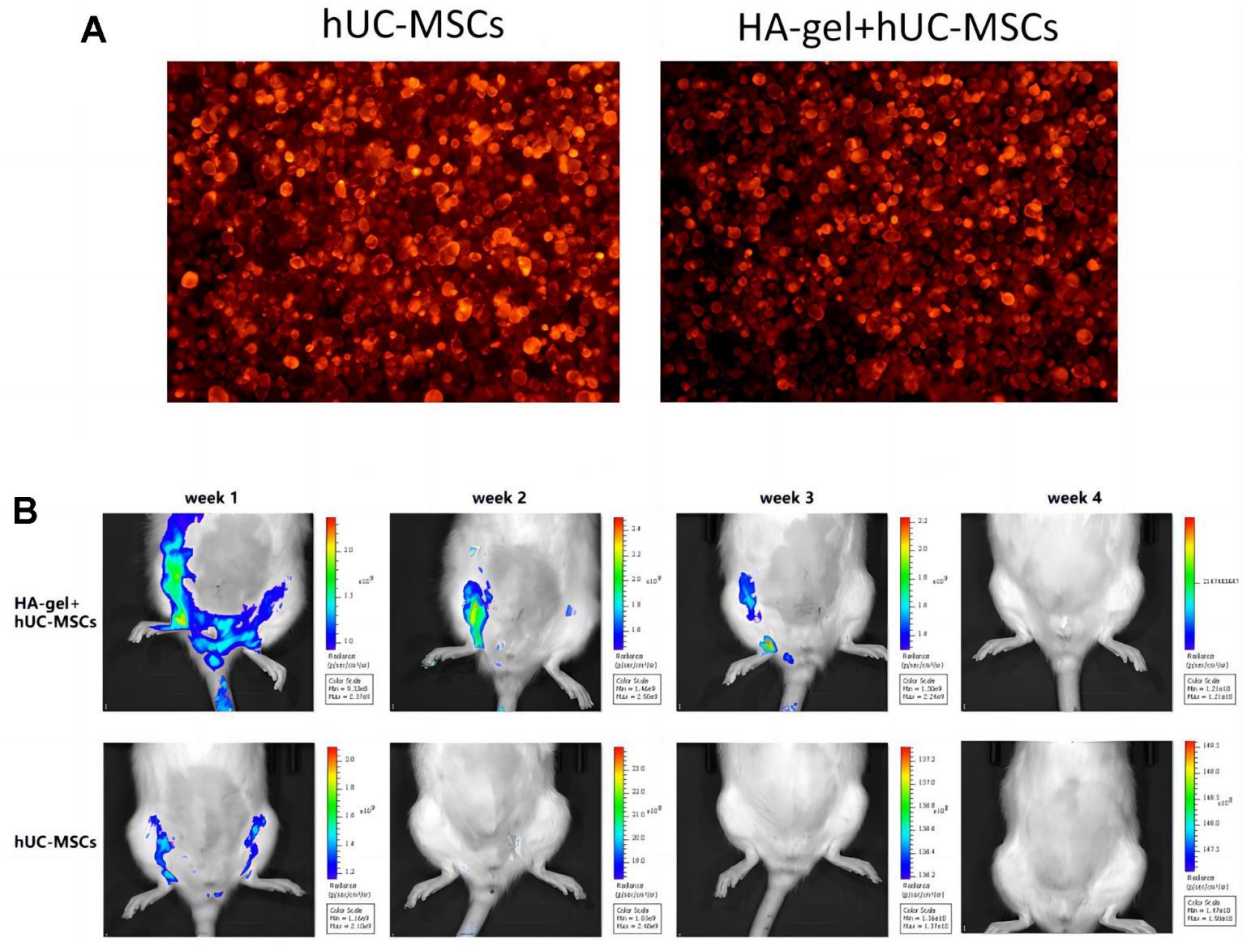
**The effect of HA-gel on hUC-MSCs in IUA model.** (**A**) The morphological differences; (**B**) The residence time of hUC-MSCs in the rat uterine endometrium.

A total of 40 rats are divided into 5 experimental groups: ① Control group (CON), ② IUA group, ③ HA-gel group, ④ hUC-MSCs group, ⑤ HA-gel + hUC-MSCs group. Each group consists of 8 rats. When compared to untreated hUC-MSCs, the morphological differences in hUC-MSCs treated with HA-gel were merely insignificant, and the cell growth status was good in both groups. The effectiveness of cell membrane labeling was evaluated using PKH26 dye, and subsequent observation under a fluorescence microscope revealed that cells from both groups showed significant staining. As shown in Figure 4A there was no significant difference in the number of stained cells between the groups. *In vivo*, imaging was used to observe the residence time of hUC-MSCs in the rat uterine endometrium and evaluate HA-gel’s supporting and fixing effect on hUC-MSCs at the uterine site. HA-gel could prolong the residence time of hUC-MSCs in the rat uterine endometrium (Figure 4B).

### Endometrial morphological structure and extracellular matrix deposition

**Figure 5:**
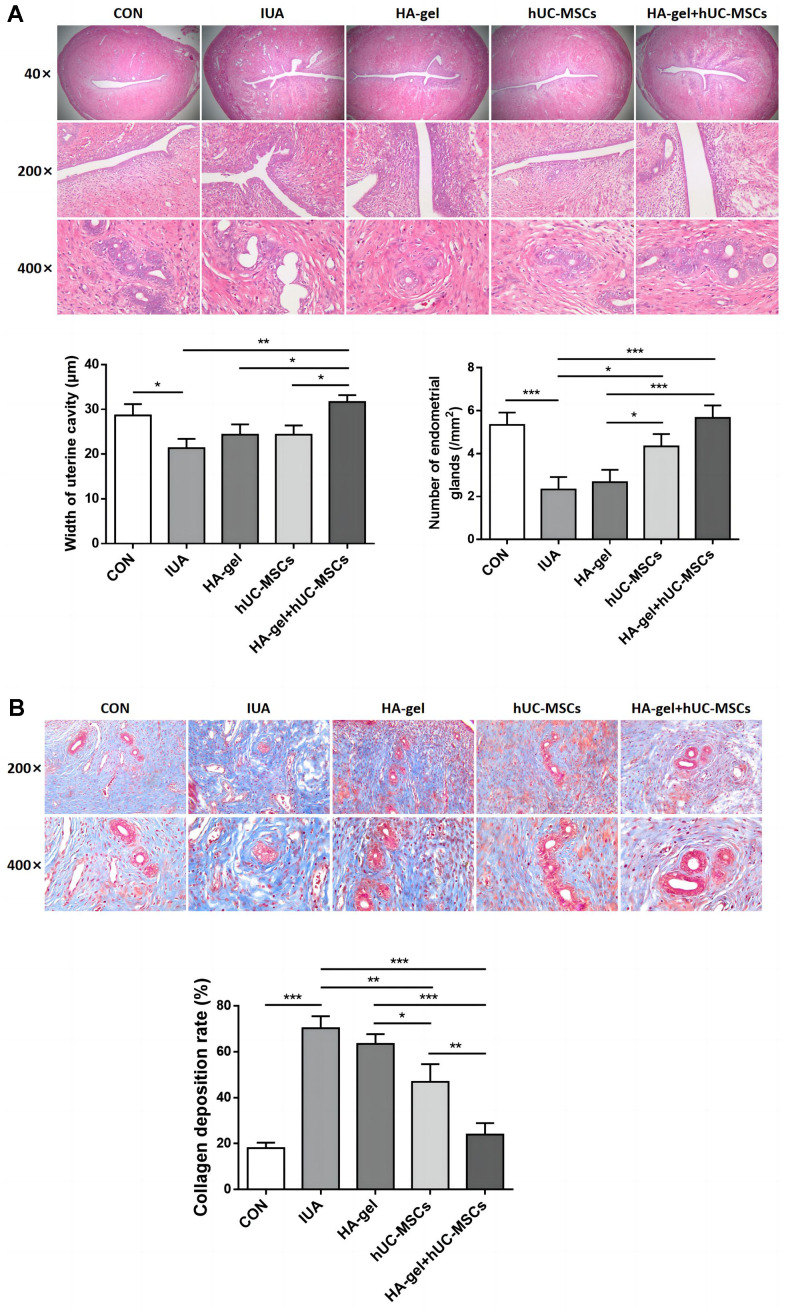
**Effect of different treatments on endometrial regeneration and collagen remodeling.** (**A**) HE stains shows the width of uterine cavity and number of endometrial glands; (**B**) Masson staining indicates the degree of endometrial fibrosis.

HE staining results showed that in the CON group, the rat uterine endometrium consisted of neatly arranged single-layer columnar epithelial cells and stroma with abundant small blood vessels. Oval or circular endometrial glands were distributed in the submucosa and basal layers, and the uterine cavity was in an open state. Compared to the CON group, the IUA group exhibited a narrower uterine cavity (P<0.01), thinner endometrium, disorganized arrangement of epithelial cells, partial glandular atrophy, and decreased glandular quantity (P<0.05). The HA-gel group demonstrated a considerably bigger uterine cavity than the IUA group (P <0.001), with no significant changes in endometrial thickness, partial glandular atrophy, or glandular quantity (P>0.05). The hUC-MSCs group on the other hand exhibited a significantly wider uterine cavity (P<0.01), thicker endometrium, partial glandular atrophy, and increased glandular quantity (P<0.05). In the HA-gel+hUC-MSCs group, there was a notable enlargement of the uterine cavity (P<0.05), alongside with a thicker endometrium. The glands were predominantly oval or circular, and there was a significant increase in glandular quantity (P<0.01). These results indicate a remarkable improvement in tissue structure (Figure 5A).

Results from Masson staining revealed that the IUA group of rats had much more collagen deposition than the CON group(P<0.001) and severe fibrosis of the uterine endometrium. Compared to the IUA group, the HA-gel group showed some improvement in uterine endometrial tissue fibrosis, but the difference was insignificant (P>0.05). Rats in both the hUC-MSCs group and the HA-gel+hUC-MSCs group exhibited a significant decrease in collagen deposition in the uterine endometrial tissue(P<0.001), while the collagen deposition in HA-gel+hUC-MSCs group was less than that in the hUC-MSCs group (P<0.001) (Figure 5B).

### Effects of different treatments therapy on endometrial proliferation, fibrosis and estrogen regulation

**Figure 6:**
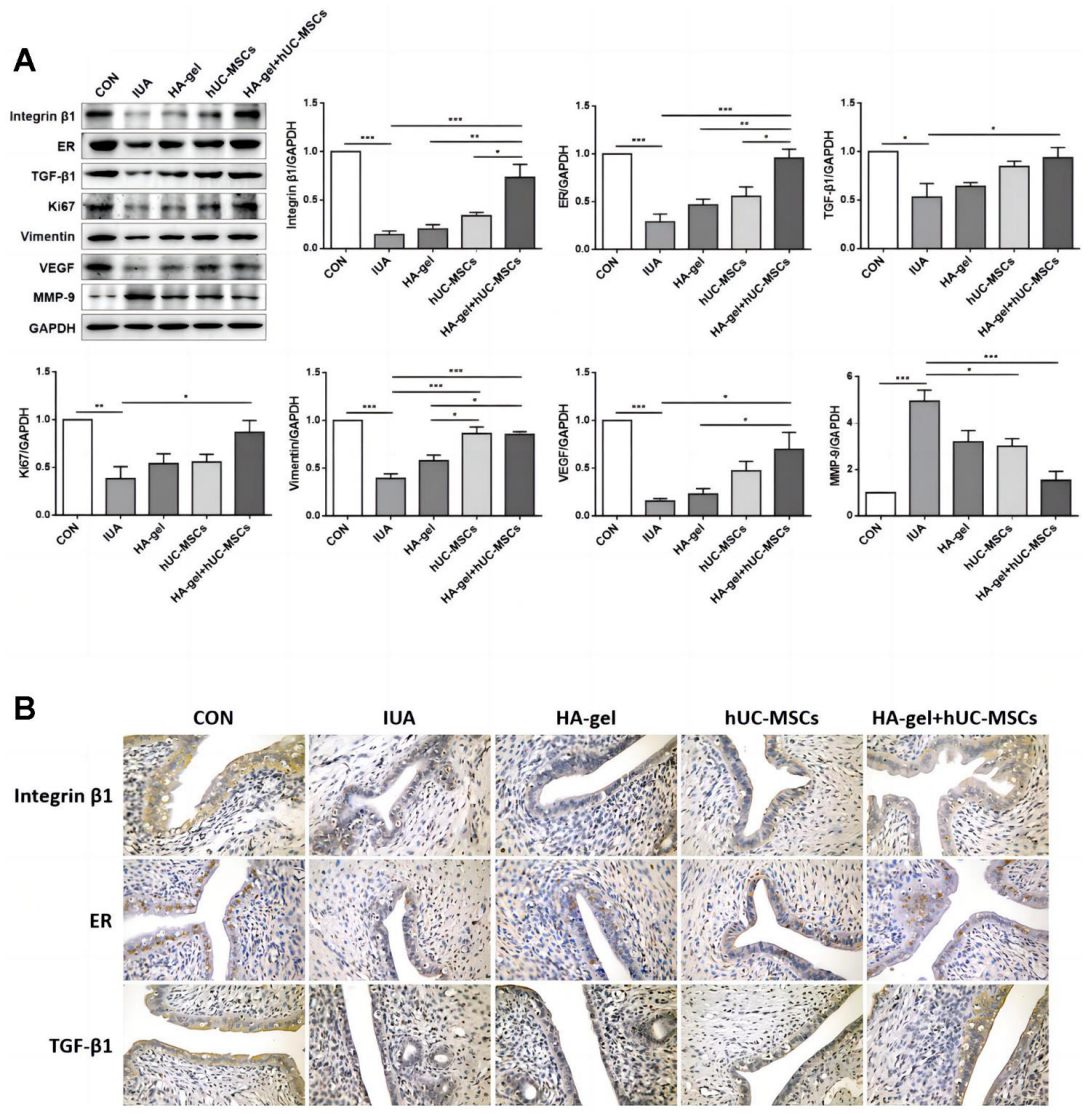
**Effects of different treatments on endometrial proliferation, fibrosis, and estrogen regulation.** (**A**) The expression levels of Integrin β1, ER, TGF-β1, Ki67, Vimentin, VEGF, and MMP-9 were determined by Western blot; (**B**) Integrin β1, ER, and TGF-β1 were confirmed by immunohistochemical analyses. RE, Estrogen Receptor; TGF, Transforming Growth Factor; VEGF, Vascular Endothelial Growth Factor; MMP, Matrix Metalloproteinase.

We further investigated the effects of HA-gel+hUC-MSCs therapy on fibrotic cytokines. Integrin β1, ER, TGF-1, Ki67, Vimentin, and VEGF were significantly upregulated, while MMP-9 was considerably downregulated in the IUA group compared to the CON group (Figure 6A). While the expression of the Integrin β1, ER, TGF-β1, Ki67, Vimentin, and VEGF was dramatically increased, MMP-9 was decreased in the HA-gel+hUC-MSCs group compared with the IUA group (Figure 6A). The HA-gel + hUC-MSCs group exhibited notable alterations in the expression of genes associated with fibrosis, estrogen, and cellular differentiation. Moreover, the IHC analyses revealed that the expression of levels of Integrin β1, ER, and TGF-β1 expression levels were consistent with those of Western blot (Figure 6B).

### Effects of different treatments on fertility restoration

**Figure 7:**
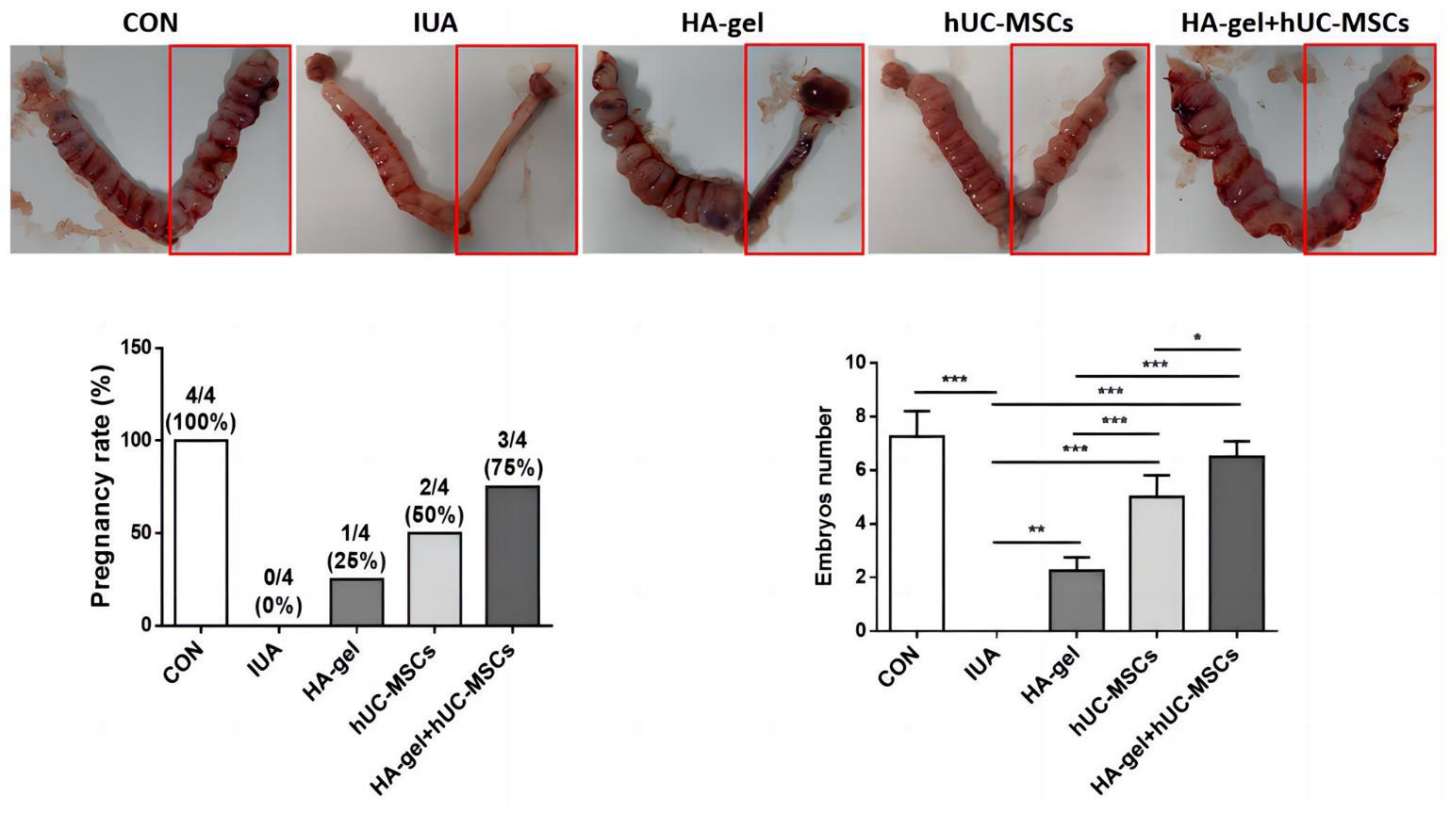
Effects of different treatments on fertility restoration.

To evaluate the effect of HA-gel+hUC-MSCs treatment on fertility restoration, pregnant uteri and the associated embryos were collected with the outcomes shown in Figure 7. The IUA group had a considerably decreased pregnancy rate and a reduced quantity of embryos compared to the CON group. However, the HA-gel+hUC-MSCs group exhibited improved fertility compared with the other groups. The findings suggest that the utilization of HA-gel + hUC-MSCs treatment has the potential to effectively regenerate a functional endometrium, therefore facilitating the effective implantation of embryos and increasing fertility restoration prospects.

## DISCUSSION

Trauma and infection can damage the endometrium’s basal layer leading to hypertrophy of fibroblasts, hypertrophy of ungulate tissues, and stimulate the formation of endometrial fibrosis, ultimately resulting in the advancement of IUA [29]. IUA is associated also with to other conditions, including amenorrhea, hypomenorrhea, secondary infertility, cyclic lower abdominal pain, and significant obstetric complications [11]. In recent years, regenerative engineering and stem cell therapy have emerged as promising and highly sought-after treatments for endometrial damage and fibrosis. Generally, the pursuit of this topic stems from the high recurrence rates observed in patients with severe intrauterine adhesions (IUA). As a result, developing an efficacious treatment for individuals suffering from IUA becomes of utmost importance. This study employed an experimental model of intrauterine adhesions (IUA) to investigate the impact of human mesenchymal stem cell (MSC) transplantation in conjunction with hyaluronic acid (HA) gel on the processes of intrauterine reconstruction and endometrial regeneration. In this study, we created an IUA model in female rats using mechanical curettage. This model was designed to mimic underlying pathological mechanism observed in patients with IUA. The findings of this study indicate that transplantation of hUC-MSCs elevated endometrial thickness, increased the number of endometrial glands, and diminished the area affected by fibrosis. Following hUC-MSCs therapy, the expression of the profibrosis molecule TGF-β1 was suppressed, leading to a reduction in collagen deposition. hUC-MSCs treatment promoted endometrial angiogenesis and proliferation while enhancing ER expression. Transplanted hUC-MSCs+HA-gel demonstrated sustained presence in the endometrium for 3 weeks. The findings from this study offer a very important and valuable direction for the future clinical trials and the development of new methodologies for the treatment.

Current knowledge holds that endometrial fibrosis, low estrogen levels, and a deficiency in endometrial stem cells are the leading causes of IUA [30]. Our study further revealed that the administration of hUC-MSCs promoted the upregulation of ER, TGF-β1, Integrin β1, Ki67, Vimentin and VEGF expressions while facilitating the suppression of MMP-9 expression in rats afflicted with IUA. The prominent observation was particularly noticeable in the case of hUC-MSCs incorporating HA-gel. Within these factors, TGF-β1 stands out as a crucial profibrogenic factor, overseeing cellular growth and repair processes. Among all the factors, TGF-β1 promotes the production of extracellular matrix, mesenchymal cell proliferation, and the accumulation of collagen and fibrin [31]. ER binds with estrogen, triggering transcriptional activation and boosting cellular DNA and protein synthesis. Additionally, it increases the proliferation of endometrial epithelial cells, demonstrating the multifaceted role of these factors in IUA prevention [32].

Ki67 is extensively utilized as a marker for cells actively undergoing proliferation. Integrin β1 is a transmembrane protein that plays a significant role in cell adhesion and signaling. It plays a part in several cellular activities, including cell migration, proliferation, and differentiation. Integrin β1 interacts with extracellular matrix proteins and mediates cell-extracellular matrix interactions [33]. Vimentin, a type III intermediate filament protein, is commonly found in mesenchymal cells and provides structural support while also playing an important role in cell integrity. Vimentin affects a variety of naturally occurring processes, involving cell migration, adhesion, and signaling [34]. VEGF is crucial for angiogenesis and tissue repair, including the endometrial lining. Disruptions in the normal VEGF signaling pathway may lead to impaired angiogenesis and inadequate tissue regeneration in the uterus, resulting in scar tissue formation [35]. Extracellular protease MMP-9, a member of the metzincin family, is essential for preserving the balance between fibrosis and antifibrosis. An excessive breakdown of the extracellular matrix and subsequently tissue fibrosis can occur when the balance of factors that both encourage, and hinder fibrosis is disrupted [36]. Hence, we suggested that HA-gel combined with hUC-MSCs could enhance cell proliferation by upregulating the expression of ER, TGF-β1, Integrin β1, Ki67, Vimentin and VEGF. Additionally, it could hinder fibrosis by suppressing MMP-9 expression in rats afflicted with IUA.

Migration of MSCs to targeted tissues is an essential process encompassing their viability and guided movement [37]. The ability of MSCs to migrate to ischemic or damaged tissues is a significant characteristic [38]. MSCs can come from both allogeneic and autologous sources. After tissue injury, MSCs tend to move from their storage sites into the circulatory system and independently target the site of damage, thereby actively participating in tissue repair. Exogenously administered MSCs can also migrate towards specific tissues or organs, exerting their reparative functions [39]. Numerous studies have provided evidence that the migratory capacity of MSCs plays crucial roles in various diseases, contributing to their therapeutic efficacy [40]. Our study revealed that the combined therapy involving hUC-MSCs with HA-gel significantly enhanced vimentin expression. These findings have far-reaching implications, implying that by integrating specific therapeutic agents or treatments, we can enhance stem cells’ natural ability to seek out and repair damaged tissues. This improved homing capability is especially important in the context of IUA, where effective regeneration of the endometrial layer is required to reverse the condition and restore fertility.

Stem cell-based therapy has received much attention as a potential and intriguing method for tissue regeneration. It holds great potential for the development of novel treatments in regenerative medicine. The embryonic mesoderm derived MSCs have the ability for multipotent differentiation. The cells derived from discarded umbilical cords have garnered attention as a potentially valuable and plentiful resource for cell-based therapeutics, owing to their ease of extraction and low immunogenicity. Numerous studies have demonstrated the potential of MSCs in repairing damaged tissues, further highlighting their significance in regenerative medicine. Firstly, MSCs migrate to the injured site and secrete cytokines, promoting angiogenesis and revascularization, ultimately accelerating wound healing [41]. Secondly, through autocrine or paracrine effects, MSCs assist in the restoration of cellular processes at the injured site [42]. Thirdly, they release regulatory substances that control the inflammatory response linked to wound healing, facilitating the healing of injuries [43]. Lastly, MSCs produce bioactive molecules that inhibit the formation of scars fostering an environment that is favorable for tissue regeneration [44]. These diverse mechanisms contribute to the therapeutic potential of MSCs in advancing treatment approaches in restoring the structure of damaged tissues and intensifying their role in regenerative medicine. According to a recent study, both HA-gel and hUC-MSCs/HA-gel complexes showed the potential to restore the IUA partly after a mechanical injury. However, the transplantation of hUC-MSCs/HA-gel complexes exhibited dual repair effects, supporting endometrial regeneration and a reliable antiadhesion property. This suggests that combining hUC-MSCs with HA-gel has an enhanced therapeutic impact on IUA by preventing adhesion formation and facilitating endometrium regeneration [45]. Although that study highlighted the partial repair effects of HA-gel and hUC-MSCs/HA-gel complexes on intrauterine adhesions, their influence on fertility recovery was not extensively explored. Our study conducted additional evaluations to assess the therapeutic impact of hUC-MSCs/HA-gel on fertility restoration. The results revealed that the treatment involving hUC-MSCs-loaded and crosslinked HA-gel significantly promoted fertility recovery in SD rats [45].

In this research work we showed that HA-gel, hUC-MSCs, and hUC-MSCs/HA-gel complexes could all partially repair severe IUA and promote the restoration of fertility caused by mechanical injury, but the dual repair effects of the antiadhesive property and stimulation of endometrial regeneration were significantly enhanced by the transplantation of hUC-MSCs/HA-gel combination. By creating a complex of hUC-MSCs/HA-gel with a biomaterial to prevent adhesion and enable stem cells to act at the right region of endometrial repair, we have created a method for treating patients with moderate to severe IUA and thin endometria caused by IUA. This novel approach, which employs the hUC-MSCs/HA-gel complex, has the potential to emerge as an essential method in future clinical research. It shows promise as an effective treatment, particularly as a cytokine-loaded gel, for moderate to severe intrauterine adhesions (IUA), providing new hope for patients suffering from this condition.
